# Biomechanical Evaluation of Force Loss and Simulated Tooth Movement in Maxillary Orthodontic Mesial Sliders: An In Vitro Pilot Study

**DOI:** 10.3390/bioengineering13080948

**Published:** 2026-08-21

**Authors:** Carolien A. J. Scheurer, Luise H. Dommack, Delia B. Rieken, Christoph Bourauel, Ludger Keilig, Mats Philipp Scheurer, Christopher J. Lux, Juliana Marie-Kristine Mielke

**Affiliations:** 1 Department of Orthodontics, Medical Faculty Heidelberg, Heidelberg University, Im Neuenheimer Feld 400, 69120 Heidelberg, Germany; carolien.scheurer@med.uni-heidelberg.de (C.A.J.S.);; 2 Zahnarztteam Hamm, Private Practice, Bockum-Hövel, 59075 Hamm, Germany; 3 Oral Technology, University Hospital Bonn, 53111 Bonn, Germany; 4 Department of Oral and Maxillofacial Surgery, Medical Faculty Heidelberg, Heidelberg University, Im Neuenheimer Feld 400, 69120 Heidelberg, Germany

**Keywords:** temporary anchorage device, TAD, molar mesialization, mesial slider, orthodontic space closure, biomechanics

## Abstract

Skeletal-anchored mesial sliders are increasingly used for molar mesialization, yet their mechanical behavior has not been compared under standardized conditions. This in vitro pilot study evaluated four slider designs (BENEfit^®^ Beneslider [BB], TADMAN Beneslider [TB], IZE Slider [IO; OrthoLIZE GmbH], Slider on Minipin [SO; OrthoLIZE GmbH]), each combined with elastic chains (-C) or NiTi springs (-S), to characterize force loss, mesial movement, and associated mechanical side effects using an experimental biomechanical measurement system. Using repeated measurements on one specimen with 1 N of applied force, three-dimensional tooth movements were recorded across 200 simulation steps per run. The sliders showed design-dependent mechanical patterns: IO sliders produced the smallest sagittal movements (IO-C: −0.84 mm [0.05], IO-S: −0.79 mm [0.02]) and the highest force loss (IO-C: 88.1% [2.1], IO-S: 89.8% [0.3]), whereas BB-S and TB-S generated larger mesialization distances (BB-S: −3.55 mm [0.53], TB-S: −3.46 mm [0.08]) with lower force loss (BB-S: 42.8% [9.2], TB-S: 21.1% [2.9]). Translational deviations remained small, while rotational effects were more pronounced. These findings represent mechanical tendencies of the tested configurations under idealized conditions and do not account for biological variability, periodontal compliance, or patient-specific factors. As such, the results cannot be generalized to clinical performance but provide preliminary reference data. Expanded investigations using multiple specimens, biological modeling, or finite element analysis will be necessary to determine how these mechanical patterns translate into clinical tooth movement.

## 1. Introduction

Orthodontic gap closure is an evidence-based treatment option with long-term success, particularly in cases of agenesis or premature tooth loss in the anterior or posterior region, offering both functional and periodontal advantages [1,2,3,4]. Closing gaps with the patient’s own teeth enables a definitive restoration to be achieved as early as adolescence. Prosthetic restorations can be avoided, along with their long-term follow-up costs [2,5,6,7].

In this context, controlled molar mesialization is gaining increasing importance in clinical practice [1,2,3,4]. In addition to its high therapeutic potential, mesial movement of the molars is also biomechanically challenging [8,9,10]. Without precisely planned mechanics, undesirable side effects such as tipping, rotation, vertical deviations, torque loss, or anchorage loss can occur, even in the anterior region [10,11,12]. Conventional anchorage concepts, for example, with intermaxillary elastics, are highly dependent on patient compliance and can also lead to undesirable reactive sagittal and vertical forces, such as retrusion of the lower anterior teeth [12,13]. Anchorage control is a key challenge, especially in cases of asymmetrical mesialization or when mesialization with maximum anchorage without reciprocal retraction of the anterior teeth is required.

The introduction of temporary anchorage devices (TADs) has significantly expanded the biomechanical possibilities of molar mesialization [14,15,16,17,18]. Direct anchoring in the bone largely eliminates reactive forces [16,17,18]. Skeletal-anchored mesial sliders thus enable non-compliance-dependent, more predictable force transmission without loss of dental anchorage and have therefore established themselves clinically as efficient appliances [3,14,15,19,20,21].

It should be noted that factors such as the distance between the point of force application and the center of resistance, the geometry of the guide bar, the coupling and play between the slider and the guide bar, as well as other connection and material properties, have a decisive influence on friction, the resulting force and moment system, and thus the three-dimensional tooth movement [3,13,22,23,24,25,26,27,28,29]. Although mesial sliders are increasingly established in clinical practice [3,4,7,15,21], to the authors’ knowledge, evidence on the three-dimensional biomechanical behavior of slider-guided molars during mesialization remains scarce [20,29,30]. In particular, comparative data on the biomechanical performance of different sliders from different manufacturers are lacking. However, a detailed understanding of the friction behavior, the forces and moments acting, and the resulting tooth movement in all six degrees of freedom is essential for knowledge about controlled tooth movements and further optimization of these appliances.

Experimental measurement and simulation systems enable precise analysis of orthodontic force systems and the resulting tooth movements under standardized conditions [31,32,33]. In this context, the present in vitro pilot study aims to analyze the force loss of the sliders and to investigate the three-dimensional force and moment system as well as the resulting movement pattern of the molars during mesialization using various commercially available mesial sliders.

## 2. Materials and Methods

The objective of this experimental in vitro pilot study was to perform a three-dimensional analysis of the biomechanical behavior of molars during slider-guided mesialization using different slider systems. The forces and moments acting on the molars and the resulting tooth movements in all three spatial planes were examined. Mesialization was simulated, and data were collected under controlled laboratory conditions using the biomechanical test setup OMSS (Orthodontic Measurement and Simulation System).

**Figure 1:**
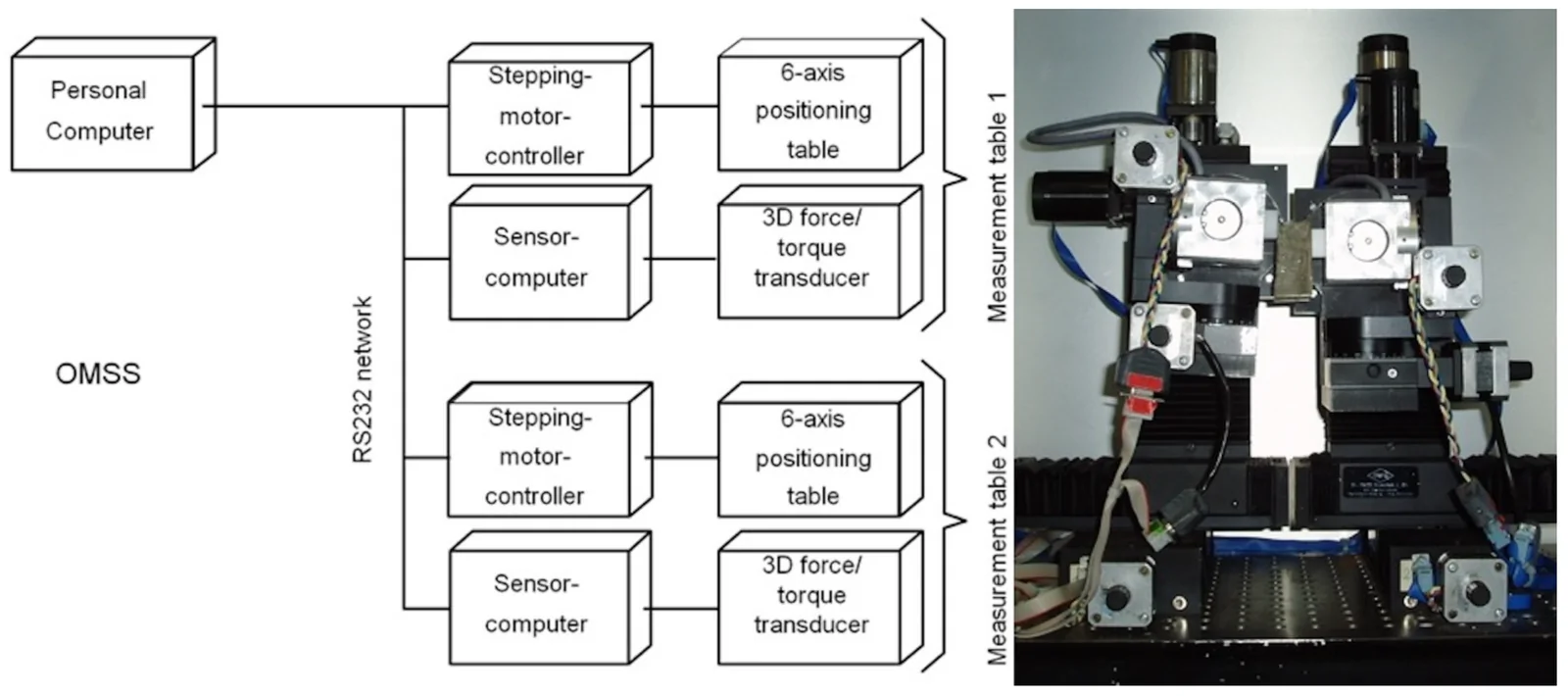
(**left**) Schematic diagram of the Orthodontic Measurement and Simulation System, consisting of measurement tables 1 and 2, each equipped with a six-axis positioning table and a 3D force/torque sensor. (**right**) Photograph of the OMSS with measurement table 1 (**left**) and table 2 (**right**). Photographs adapted from Bourauel et al. 1992 and Drescher et al. 1991 [30,31].

The OMSS is a measurement system specially developed for simulating orthodontic tooth movements. Figure 1 shows a schematic diagram of the measurement setup (left) and an overall view of the OMSS in its temperature test chamber (right). It consists of two independently controllable measuring tables, having six degrees of freedom each and an integrated 6-axis force/torque transducer [31]. The sensors have been calibrated for a measuring range of ±15.0 N for forces and ±450 Nmm for moments, with a resolution of 0.02 N for forces and 0.5 Nmm for moments, respectively. Accuracy of forces and moments was determined to be 0.02 N and 0.09 Nmm, respectively [30]. The data were recorded using specially developed software. The design and validation of the system have been described in detail in previous publications [30,31].

**Figure 2:**
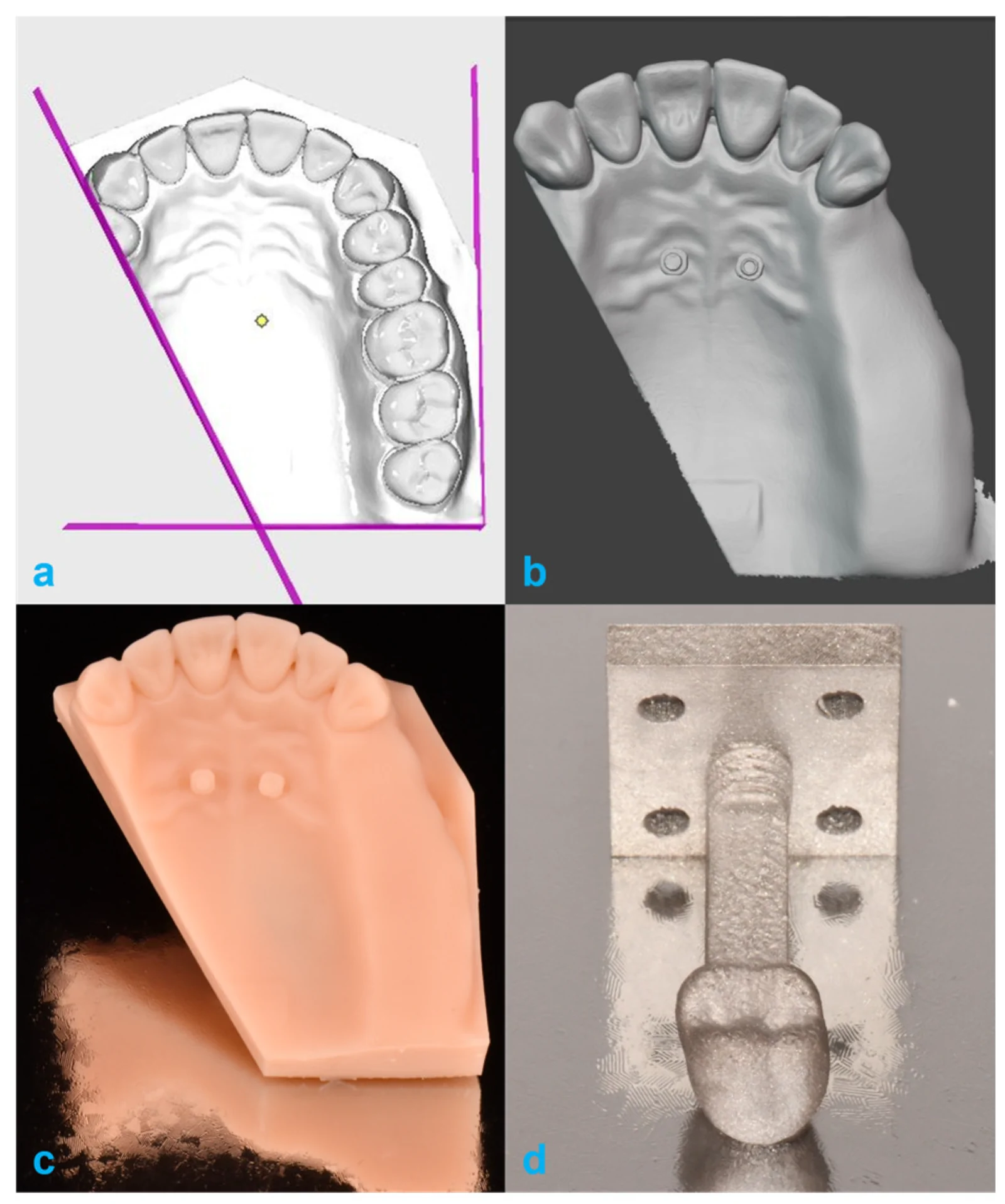
Manufacturing process for the models and the tooth to be moved. (**a**) Trimming of the model along purple lines (OnyxCeph^3^™, Version 3.2.68(74), Image Instruments GmbH, Chemnitz, Germany); (**b**) Final shaping of the model (Blender, Version 3.6.1, Blender Foundation, Amsterdam, The Netherlands); (**c**) Model printing (Model V3, Formlabs 3b, Formlabs GmbH, Berlin, Germany); (**d**) Metal printing of the tooth with a mount for sensor 2 (Maraging-Steel M300, PROTOTEC GmbH & Co. KG, Attendorn, Germany).

A standardized upper jaw teaching model (Frasaco AG-3 OK, Frasaco GmbH, Tettnang, Germany) was digitized and the teeth segmented so that the first upper molar could be digitally isolated (OnyxCeph^3^™, Version 3.2.68(74), Image Instruments GmbH, Chemnitz, Germany; Blender, Version 3.6.1, Blender Foundation, Amsterdam, The Netherlands) (Figure 2a). To enable the placement of the test specimen and its movement via the OMSS sensor, the premolars and molars were digitally removed from the model (Figure 2b). The model was additively manufactured (Modell V3, Formlabs 3b, Formlabs GmbH, Berlin, Germany) and, after insertion of two paramedian mini-implants (BENEfit^®^, PSM Medical GmbH, Gunningen, Germany) as skeletal anchors, digitized again (Figure 2c). The upper molar was modified as a test specimen for sensor connection for the OMSS and printed from metal (Maraging-Steel M300, PROTOTEC GmbH & Co. KG, Attendorn, Germany) (Figure 2d).

**Figure 3:**
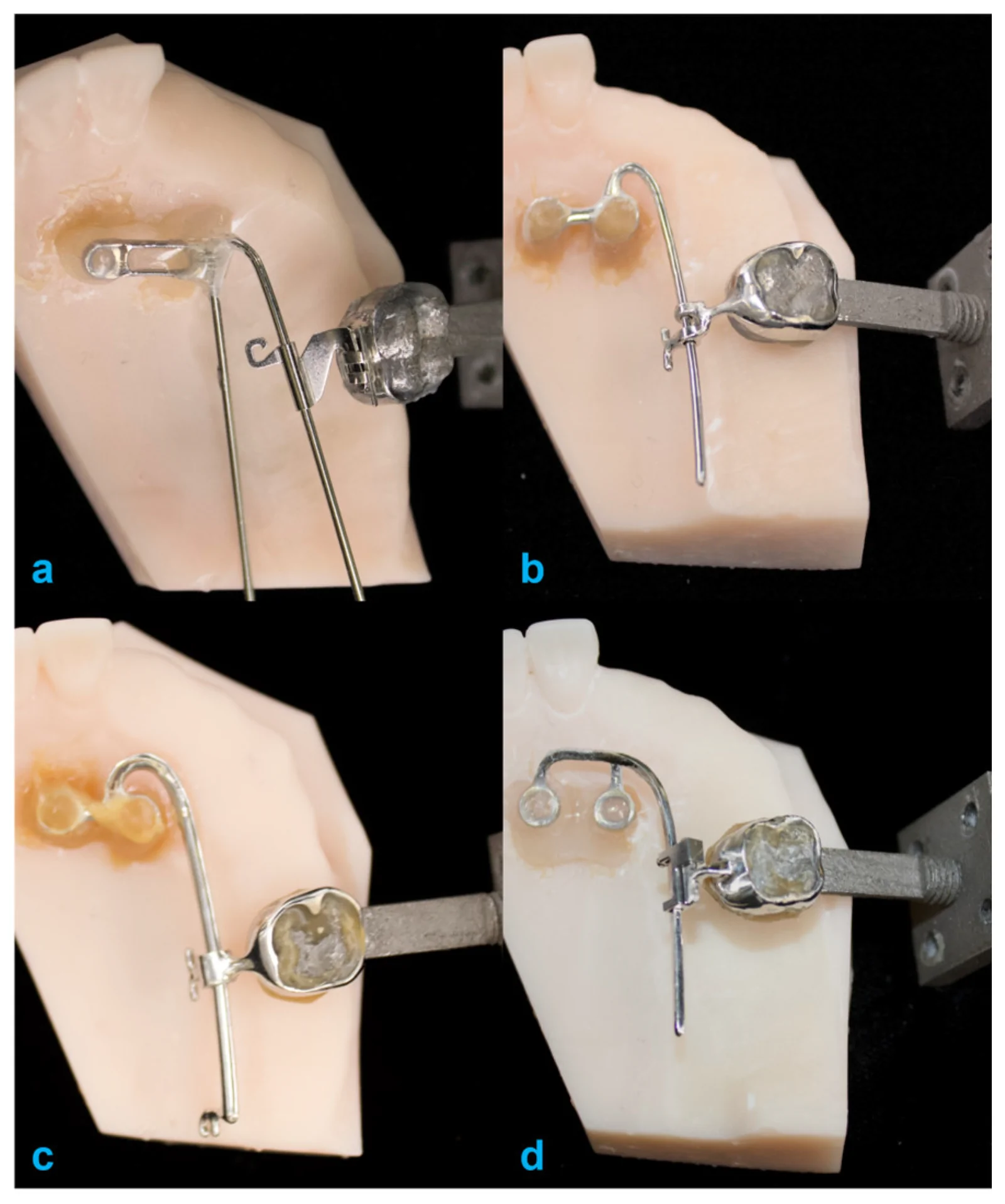
Different sliders attached to the test model with a test molar. (**a**) BB slider; (**b**) SO slider; (**c**) IO slider; (**d**) TB slider.

To order precisely fitting CAD/CAM mesial sliders, the isolated test molar was digitally placed in the correct position. The hand-bent slider was fitted and secured directly onto the test model (Figure 3). Table 1 shows the mesial slider systems examined with different configurations between the molar band and the guiding arch. The dimensions of the guiding bars used in the tested slider configurations were measured.

**Figure 4:**
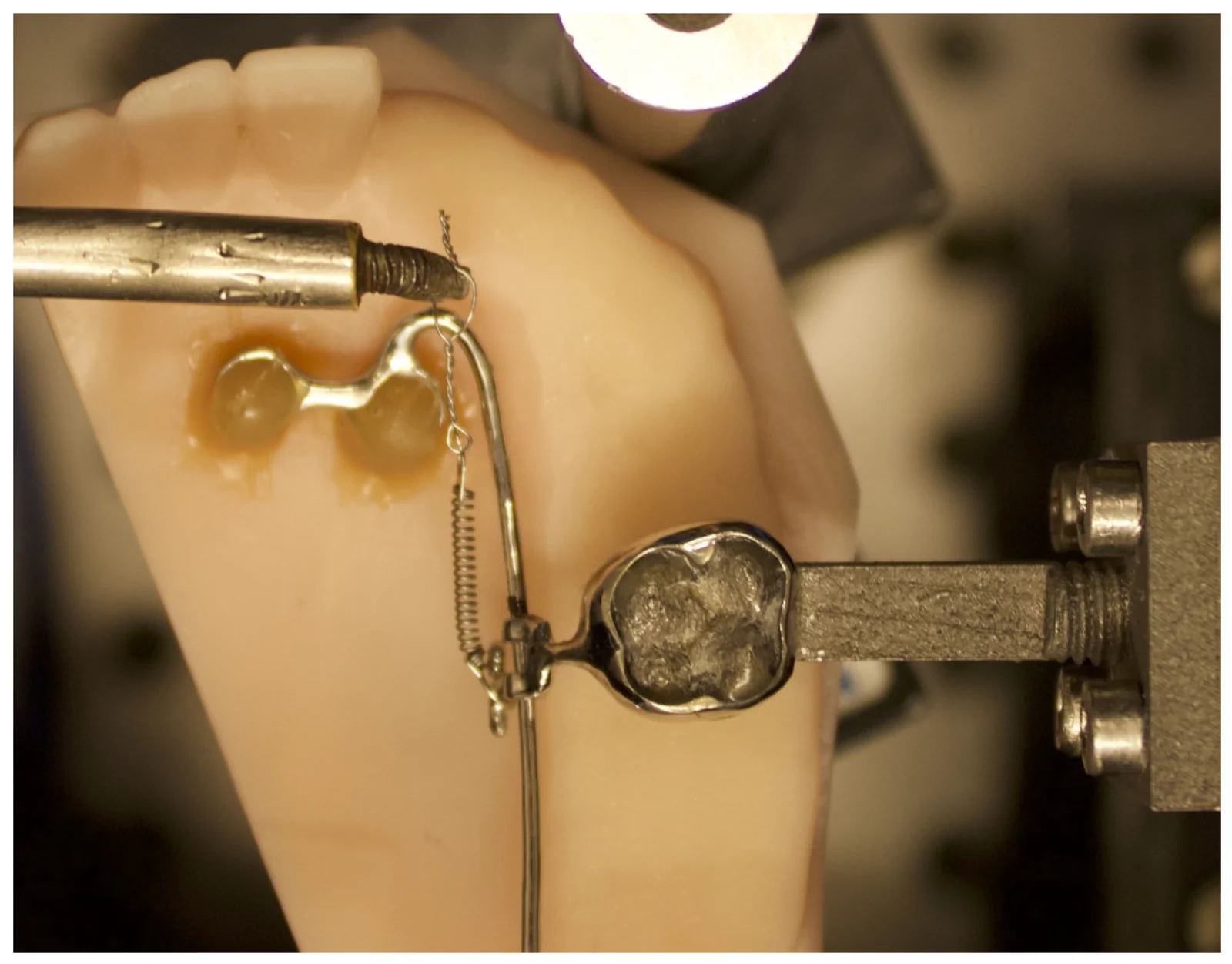
Model secured in the OMSS using a NiTi spring and SO slider.

The entire setup was mounted in the OMSS (Figure 4). Both elastic chains (-C; Gray Generation II Power Chain™, Ormco Corporation, Brea, CA, USA) and NiTi pull springs (-S; NiTi Closed Coil Spring with Eyelets, 9 mm, heavy; dentalline GmbH & Co. KG, Birkenfeld, Germany) were used as active elements for mesialization. A total of eight different configurations were analyzed: SO-C, SO-S, IO-C, IO-S, TB-C, TB-S, BB-C, and BB-S.

One end of the active element was attached to the respective slider, and the other to OMSS sensor 2. The initial activation force was set to F = 1 N. No reactivation was performed during the subsequent 200 simulation steps, and the force was therefore not maintained at a constant level but allowed to change according to the respective force system and the ongoing tooth movement. After activation via sensor 2, forces and moments were recorded at sensor 1 in 200 steps in six degrees of freedom. The forces and moments acting on the molar were converted into orthodontic tooth movement using the mathematical OMSS model [30]. This calculated movement was executed via the system’s stepping motor-driven axes. To represent complex three-dimensional tooth movements, the simulation process was carried out in incremental steps. Each individual simulation step consisted of three phases: Measure force system—Calculate movement—Execute movement. Force systems are measured at the point of force application, i.e., at the center of the slider in the guiding bar. Taking the position of the center of resistance on the long axis of the tooth and with a vertical distance of 6.5 mm from the level of the guiding bar in the direction of the apex [33], the force system of each individual step was transformed into a force system at the center of resistance. A transformation matrix was then calculated from the three forces and moments and incremental translational and angular movements [30]. The transformation matrix is described in [30]. For each slider configuration, 10 repeated runs were performed using the same physical test model. The slider remained fixed to its respective model throughout all 10 runs. A new physical model was used for each slider system. After completion of all measurements for one slider design, the test molar was transferred to the next slider-specific test model. Because the slider was not removed between repeated runs and the test molar was manufactured from metal, relevant mechanical wear during the repeated measurements was not expected. The order of testing was not formally randomized but followed the practical sequence of the prepared setups.

Since all materials except the NiTi springs were not temperature-sensitive, the experiments were conducted at room temperature (21 ± 1 °C). Since the austenite finish temperature of the NiTi spring was around 20 °C, this was acceptable for the spring as well; an increase in temperature to 37 °C would merely have required a lower level of spring activation. Furthermore, all tests were conducted without lubrication—such as artificial saliva—since the body of research regarding the influence of lubrication is highly contradictory, and the majority of friction studies are performed under dry conditions [34].

The force losses within the respective slider systems were calculated from the ratio of the force applied to the slider (sensor 2) to the orthodontically effective force on the molar (sensor 1) and defined as a percentage force loss (according to the following Equation (1) [35,36]:

Force loss (%) = [1 − (F_effective_/F_applied_)] × 100). (1)

Due to the exploratory nature of this pilot study and the lack of reliable prior information on effect sizes, no formal a priori power analysis was performed. Ten repeated runs per slider configuration were performed based on preliminary testing and established experimental practice, with the number of runs chosen to assess measurement repeatability under standardized experimental conditions.

Median values and interquartile ranges (IQR) for force loss and maximum translational and rotational movements were calculated for all slider designs due to the non-normal distribution. The Kruskal-Wallis test was performed to investigate differences between all eight slider groups in terms of the force source, mean force loss, and translational and rotational movements. In the case of significant abnormalities, pairwise non-parametric comparisons were performed using Dunn post hoc tests with Benjamini-Hochberg correction. For each outcome variable, the 28 pairwise comparisons among the eight configurations were treated as a separate outcome-specific family, and the Benjamini-Hochberg correction was applied within each family. A significance level of *p* < 0.05 was set for all analyzes. Statistical significance should be interpreted within the context of measurement reproducibility of the single experimental setup and does not imply population-level differences or biological variability. Statistical evaluation and the creation of graphics were performed using R (version 4.5.2) in RStudio (Posit PBC, Boston, MA, USA).

## 3. Results

### 3.1. Descriptive Statistics

The descriptive statistics showed differences in force loss, sagittal translation, and tipping between the eight slider designs (Table 2). The BB and TB sliders achieved the largest mesialization distances, whereas the IO sliders exhibited the highest resistance to sliding. The most pronounced rotational side effects were observed in the BB slider configurations.

### 3.2. Comparison of All Eight Slider-Force Combinations

The Kruskal-Wallis test revealed significant differences between the eight slider configurations for all clinical parameters evaluated. Force loss (Chi^2^ = 76.02, *p* < 0.0001) and mesialization (Chi^2^ = 72.73, *p* < 0.0001) differed significantly between the sliders. This suggests that the geometry of the slider influences not only the force transmission but also the efficiency of mesial translation. In addition, the translational side effects showed clear group-specific differences. Transverse translation (Chi^2^ = 66.91, *p* < 0.0001) and vertical translation (Chi^2^ = 59.66, *p* < 0.0001) varied significantly between slider configurations. Similarly, significant differences were observed in all three tipping and rotation parameters (maximum crown tipping: Chi^2^ = 74.02, *p* < 0.0001; maximum crown torque: Chi^2^ = 69.85, *p* < 0.0001; maximal crown rotation: Chi^2^ = 64.01, *p* < 0.0001). These results suggest that each slider configuration has a distinct biomechanical side effect profile that affects sagittal, vertical, and rotational control.

### 3.3. Force Loss

**Figure 5:**
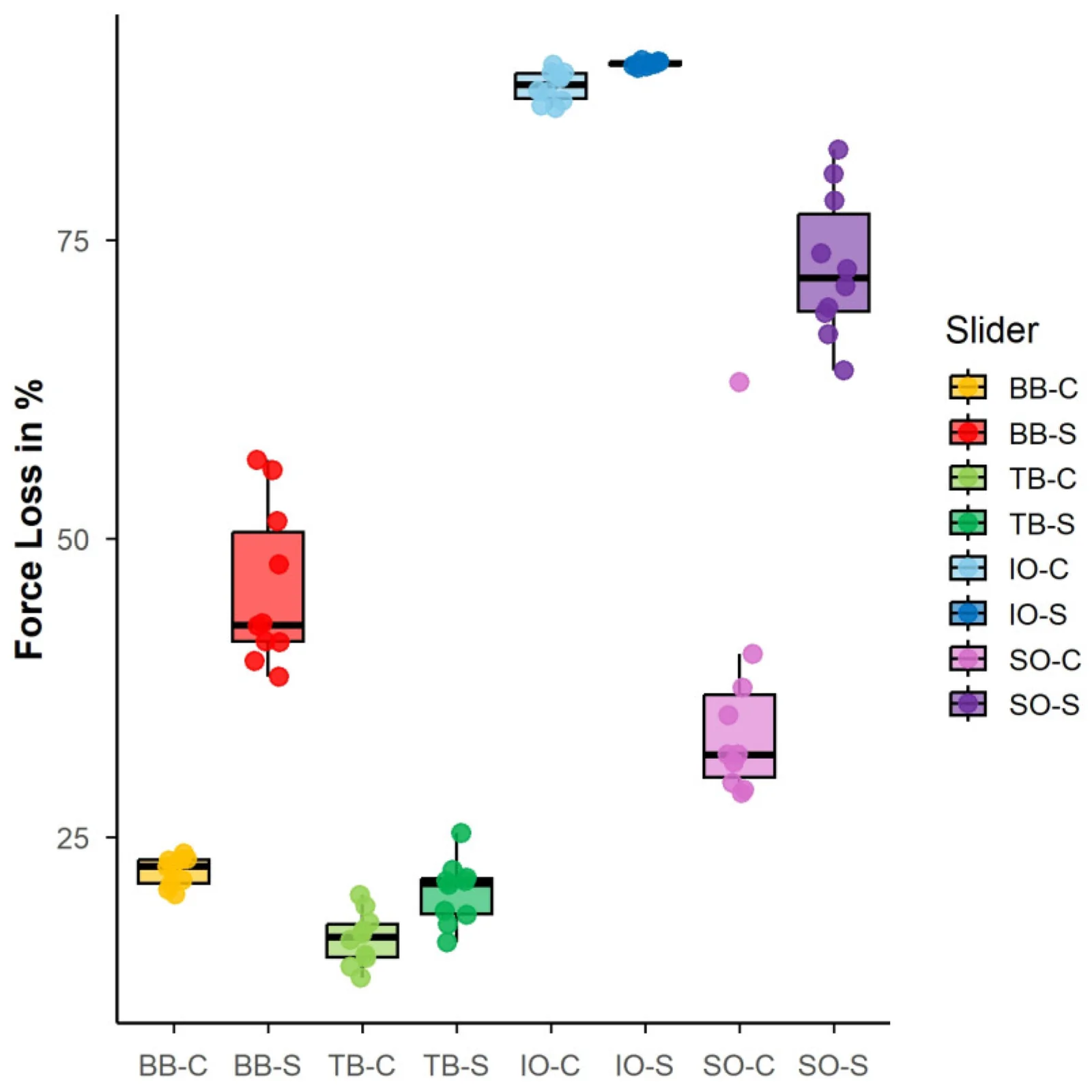
Box plot of the mean force loss in % for all slider configurations. Shown are the mean force loss values calculated over 200 simulation steps per run. Ten runs per slider. Box-and-whisker plot.

The Dunn post hoc analysis revealed a consistent pattern: BB-C, TB-C, and TB-S showed similarly significant differences compared with the IO and SO variants. TB-C and TB-S were comparable to one another but showed clear differences from both IO configurations. IO-C and IO-S formed a homogeneous group, which differed clearly from several BB and TB variants. SO-C and SO-S showed the greatest differences between themselves (Table 3). This resulted in stable, distinct friction groups with clear differences between the several types of sliders. Mean force loss was generally lower when using an elastic chain (Figure 5).

**Figure 6:**
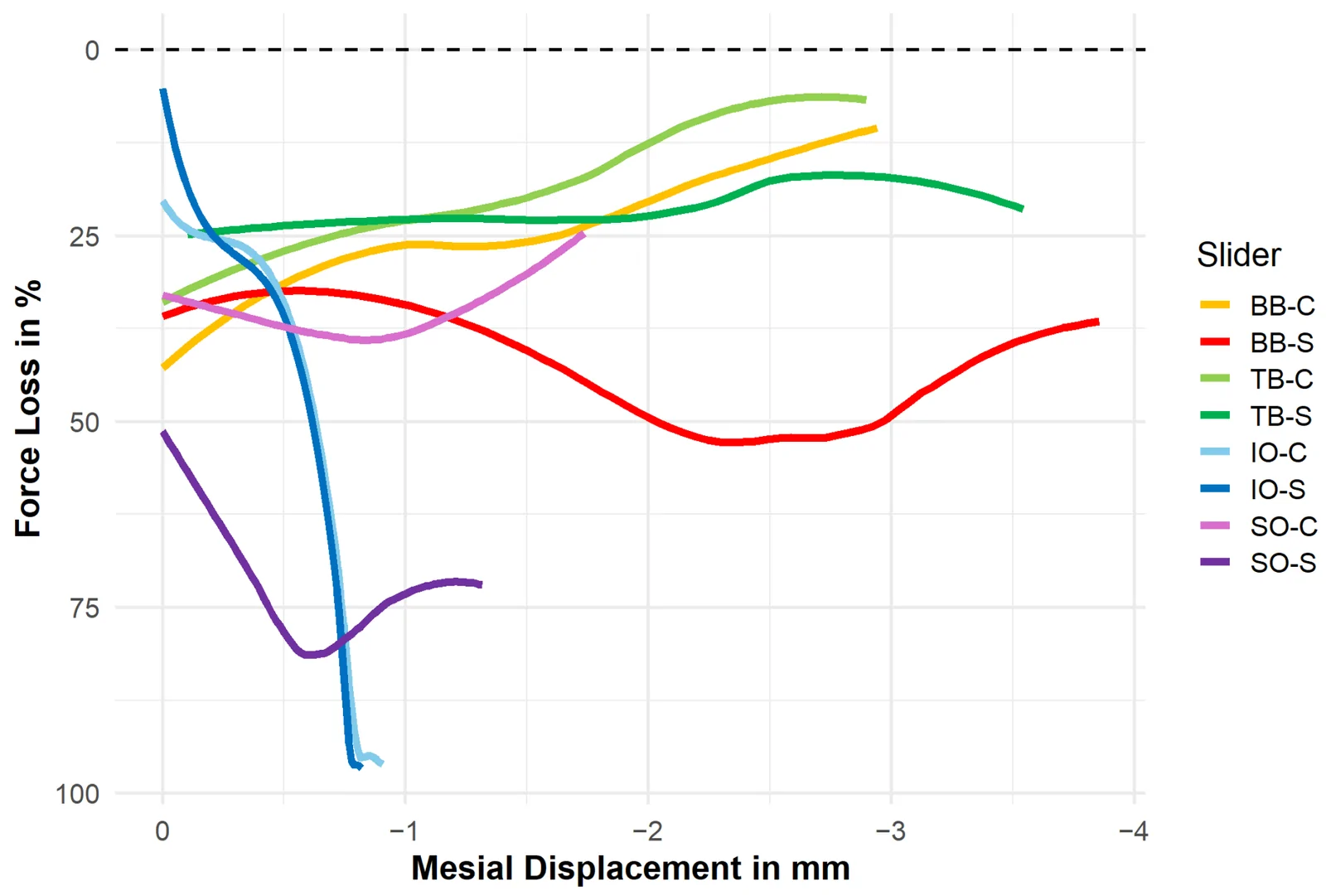
Force loss as a function of mesial displacement for all slider configurations. Individual force-loss trajectories are shown across the simulated range of mesial displacement. The curves illustrate the progression of force loss throughout the movement and allow comparison of the force-transmission behavior between slider configurations.

To characterize the force-loss behavior across the entire simulated range of motion, force loss was additionally plotted as a function of mesial displacement (Figure 6). The configurations showed distinct and non-linear force-loss trajectories. The IO and SO configurations exhibited the most pronounced increases in force loss, reaching approximately 75–95% at their respective maximum displacements, whereas most BB and TB configurations remained within a lower force-loss range.

### 3.4. Mesialization

**Figure 7:**
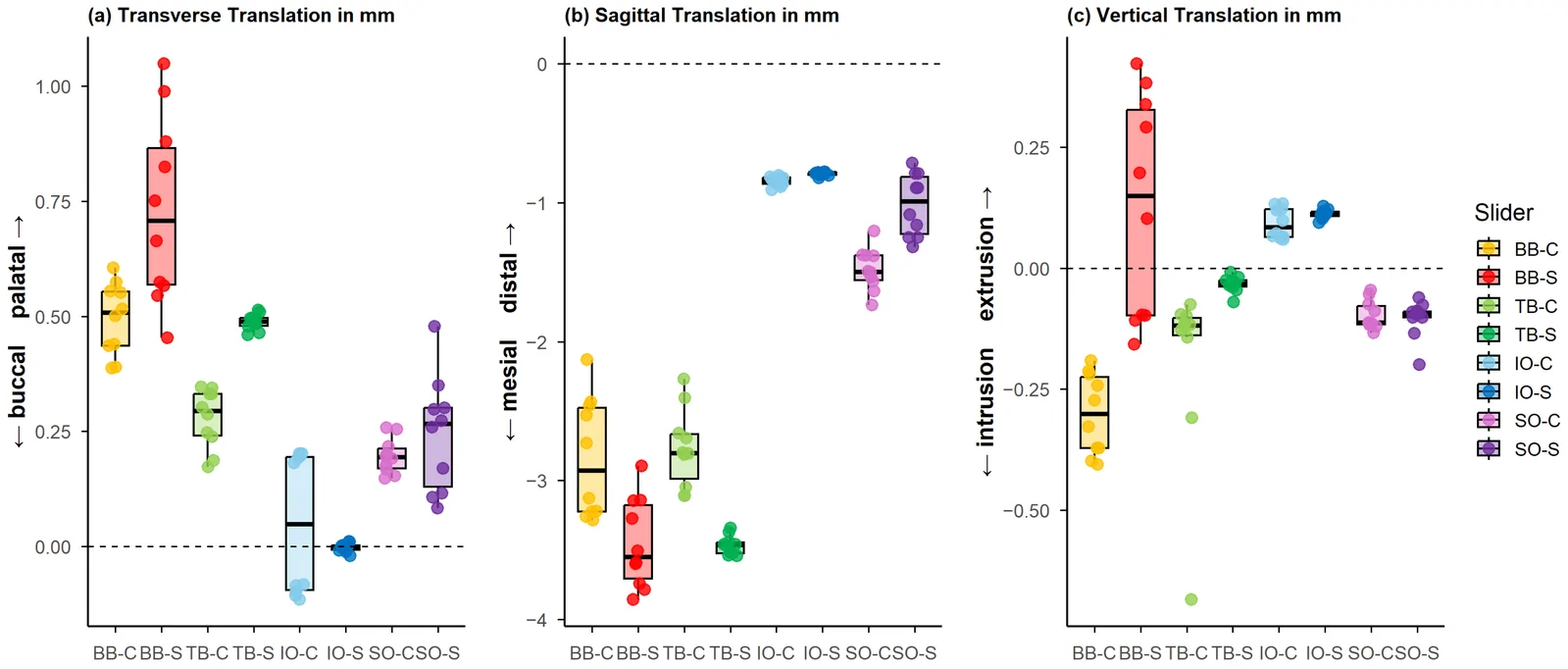
Box plot of the maximum translational movements in mm at step 200 for all slider configurations. (**a**) Transverse displacement in bucco-palatal direction; (**b**) sagittal displacement in mesio-distal direction; (**c**) vertical displacement with intrusion and extrusion. Box-and-whisker plot.

The Dunn post hoc analyses showed significant differences in maximum mesialization between several slider configurations (Table 4). Mesialization was significantly greater with the BB and TB sliders than with the IO sliders. In addition, BB-S and TB-S showed significantly higher mesialization than SO-C and SO-S. In contrast, differences between BB and TB variants were not significant. The pairwise comparisons confirmed that BB and TB sliders produce the greatest mesialization, while IO sliders achieved the least mesialization (Figure 7b).

### 3.5. Translational Side Effects

In the transverse direction, the slider configurations showed significant differences. The strongest deviations occurred between the TB-S and BB sliders and the IO sliders, which were confirmed by significant results. In particular, the comparisons with IO-C and IO-S consistently showed small adjusted *p*-values (Table 5). IO sliders have low transverse side effects overall, while BB configurations have the strongest side effects. All slider configurations, except for the IO slider, showed a trend towards palatal deflections (Figure 7a).

The analysis of vertical translations showed clear group-specific differences. The deviations between BB-C and TB-C and the IO groups were particularly pronounced, showing multiple significant effects (Table 6). Overall, the pattern suggests that the IO sliders produce different vertical side effects, with a trend towards extrusive movement compared with the other groups. BB-S showed both intrusive and extrusive deflections (Figure 7c).

### 3.6. Rotational Crown Changes

**Figure 8:**
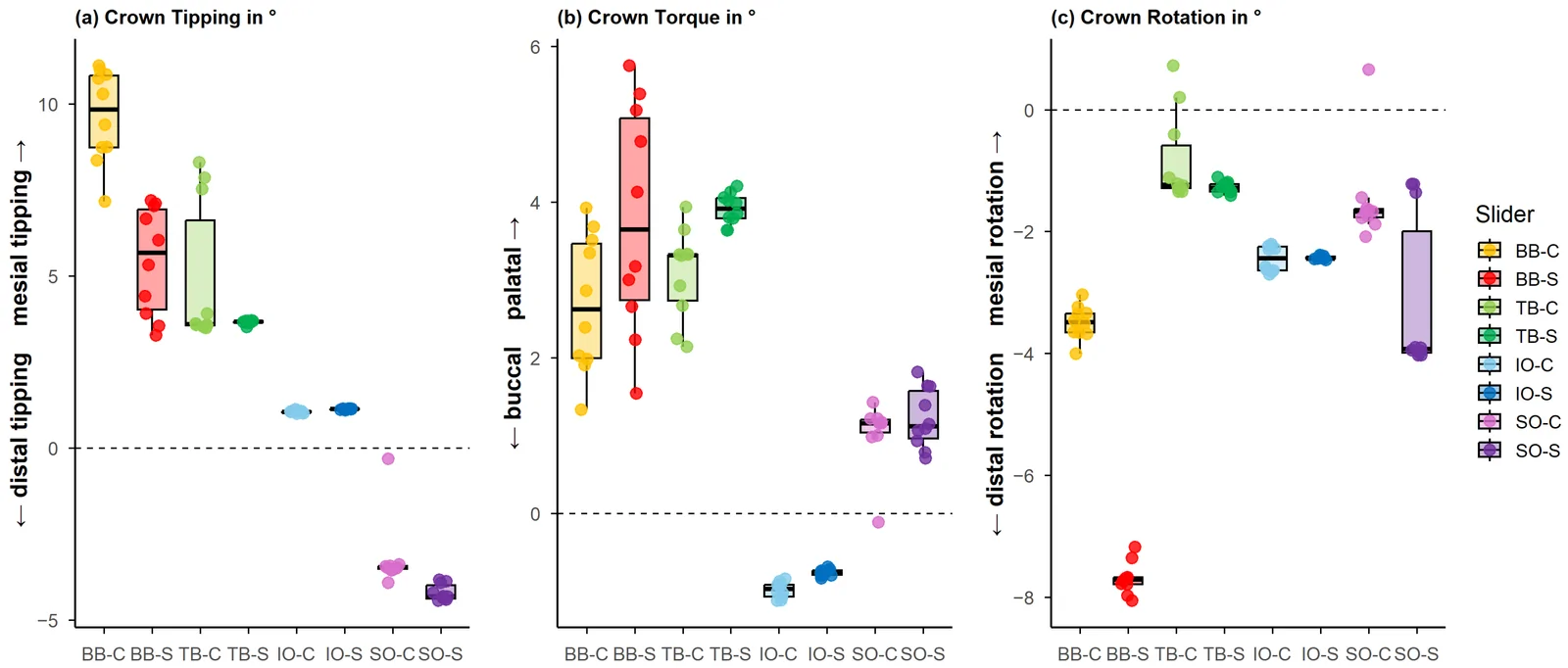
Box plots showing the maximum rotational changes in ° at step 200 for all slider configurations. (**a**) maximum crown tilt; (**b**) maximum crown torque; (**c**) maximum crown rotation. Box-and-whisker plot.

The Dunn post hoc analyses showed significant differences in crown tipping in the mesio-distal direction between the slider configurations (Table 7). All sliders except SO-S and SO-C showed distal tipping of the crown, while SO-S and SO-C showed mesial tipping (Figure 8a). BB-C showed significantly higher tipping than IO-C, IO-S, SO-C, and SO-S. BB-S, TB-C, and TB-S also showed significantly higher tipping than SO-C and SO-S, but no differences compared to BB-C. The IO sliders were in the lower range and did not differ from each other.

Significant differences were observed in the analysis of maximum crown torques between the slider designs (Table 8). Positive values indicated palatal rotations and negative values indicated buccal rotations (Figure 8b). All slider configurations showed palatal rotations, except for IO-C and IO-S, which rotated buccally. BB-C, BB-S, TB-C, and TB-S generated significantly higher crown torques than both IO variants and showed significant differences compared to the SO sliders.

When examining the analysis of maximum crown rotation, significant differences were observed between individual slider configurations (Table 9). BB-S produced the highest distal rotations and differed significantly from all other sliders, except BB-C (Figure 8c).

## 4. Discussion

The experiment conducted using the OMSS was designed to systematically record the biomechanical characteristics of the different slider configurations under standardized conditions and to simulate the resulting tooth movements [31]. The defined positioning of the sliders, fixed axes of rotation, and repeated measurements with comparable force application enabled a precise assessment of how each configuration behaved in this specific setup. The separate analysis of chain and spring configurations and the different slider designs allowed the mechanical influence of both the force applied and the design to be examined in detail. The subject of this study was to examine four different slider designs in terms of their biomechanical properties during mesial movement within one defined experimental configuration. The results reveal consistent, design-related differences in mechanical behavior.

The analysis of force loss revealed distinct patterns between the slider designs. BB variants, SO-C, and the TB variants showed the lowest force loss, whereas IO sliders and SO-S showed higher sliding resistance. The displacement-dependent analysis revealed that these differences were not expressed as uniform, linear changes across the range of motion. Instead, the slider configurations showed distinct and non-linear force-loss trajectories (Figure 6). The particularly pronounced increase in force loss observed for the IO and SO configurations suggests that their higher sliding resistance was maintained or intensified over substantial portions of the simulated displacement, whereas most BB and TB configurations remained within a comparatively lower force-loss range. The non-linear trajectory observed for BB-S, with an increase followed by a decrease in force loss at greater displacement, further indicates that the mechanical resistance of a slider may vary with its position within the guide rather than remaining constant throughout the movement. BB-C, SO-C, and the TB sliders held their position as the mechanisms with the lower force loss, indicating lower lateral contact pressure and greater tolerance between the guiding arch and slider. The markedly higher force loss observed in the IO configurations may be related to their more constrained guidance geometry. A reduced clearance or more closely coupled interaction between the slider and guiding bar may increase contact between the components and thereby increase resistance to sliding. The very low mesio-distal crown tipping observed for IO-C and IO-S is consistent with a mechanically constrained system in which increased guidance resistance may restrict both sliding and tipping. However, this does not imply that low tipping causes the high friction. Binding provides a plausible additional explanation for force loss when tipping occurs within a constrained guiding system. Previous work has demonstrated that increased tipping angles can increase resistance to sliding through binding effects [36,37]. This relationship is particularly relevant to the present findings because the configurations with lower force loss, particularly BB-C and BB-S, also permitted greater mesio-distal crown tipping, whereas the highly resistant IO configurations showed only approximately 1° of tipping (Table 2). This may indicate a bidirectional relationship, whereby constrained geometry increases contact and resistance, while tipping under load may further promote binding. Thus, the present findings are compatible with a mechanical interaction between guide constraint, contact, and tipping rather than with a simple relationship in which either friction or tipping alone determines the observed behavior. However, binding and contact forces were not directly measured in the present study, and this explanation therefore remains inferential rather than directly demonstrated.

The differences in force losses were directly reflected in the maximum mesialization distance. BB and TB sliders allowed the greatest mesial movement distances, while IO sliders and SO-S allowed the smallest. SO-C was ranked in the middle. The results thus confirm the importance of force transmission as a limiting factor for translational efficiency [27,35,36,37,38].

In the transverse plane, the BB and TB sliders showed the strongest palatal deviations, while the IO sliders showed the least lateral deviations. These differences may reflect the balance between mechanical freedom and guidance constraint within the slider systems. The greater freedom may facilitate mesial sliding but also permit larger off-axis components of the force system. Vertically, the IO sliders showed a slight extrusive tendency, while BB and TB sliders reacted predominantly intrusively or mixed. However, the absolute deviations remained low overall, which is consistent with clinical observations [20,28,29].

The pronounced mesio-distal crown tipping observed with BB and TB sliders, compared with approximately 1° for the IO configurations, may likewise reflect differences in mechanical constraint. The SO variants showed a special feature, as they were the only ones to show a tendency towards distal crown tipping. This may be related to the specific spatial relationship between the active element, guiding arch, and molar in the SO design.

In terms of crown torque, BB and TB sliders generated the strongest palatal rotations, while IO sliders were the only ones to show a buccal tendency. The larger rotational responses observed particularly in the BB and TB configurations may similarly reflect greater mechanical freedom and differences in the spatial relationship between the applied force and the tooth’s center of resistance. In configurations with less restrictive guidance, small differences in the line of force can generate a larger moment around the relevant rotational axis. Rotation around the longitudinal axis showed an independent pattern and did not correlate significantly with either friction or mesialization. This suggests that this parameter is influenced more by the individual force vector position than by the sliding mechanism.

Although the observed transverse and vertical movements were small relative to the simulated sagittal displacement or rotational movements, the present experimental design does not permit definition of a clinically relevant threshold. The numerical values should therefore primarily be interpreted as comparative mechanical characteristics of the tested configurations rather than as direct predictions of clinically relevant tooth movement.

From a clinical perspective, these findings suggest a potential trade-off between mechanical efficiency and three-dimensional control. Configurations with lower resistance to sliding, such as BB and TB, permitted greater simulated mesial displacement, but this greater mechanical freedom was accompanied by larger side effects. Conversely, the highly constrained IO configuration showed very low tipping and transverse displacement but also exhibited very high force loss. Thus, a slider design that minimizes friction is not necessarily optimal when control of unwanted tooth movements is the primary treatment objective, while a highly constrained design may improve control of certain side effects at the expense of force efficiency. This trade-off may be relevant to clinical decision-making when selecting a slider according to the intended biomechanical objective. However, these considerations should be regarded as hypotheses for clinical application rather than clinical recommendations, because the present in vitro study does not establish whether the measured differences translate into clinically relevant treatment effects.

Several limitations should be considered when interpreting these findings. First, the study used a single physical test molar and repeated measurements within one standardized experimental setup; therefore, the data characterize the mechanical response of this single configuration rather than biological variability. Second, the OMSS represents an idealized in vitro environment and does not reproduce periodontal compliance, biological adaptation, saliva, intraoral temperature, or clinical handling variability [36,38,39,40]. The experiments were also performed over a defined and relatively short activation range without repeated clinical reactivation or long-term exposure and therefore do not account for potential material fatigue or time-dependent changes in mechanical behavior. Finally, the observed translational and rotational movements represent the mechanical response of the tested configurations under the defined boundary conditions and cannot be used to establish clinically relevant thresholds or predict treatment outcomes. Future multi-specimen studies and finite element analyzes incorporating clinically relevant material properties, contact conditions, and physiologically representative boundary conditions could further elucidate the mechanisms underlying these design-dependent differences. Clinical studies will ultimately be needed to determine whether these mechanical tendencies translate into differences in force transmission and tooth movement in vivo.

## 5. Conclusions

The present pilot study identified distinct mechanical characteristics among the tested maxillary mesial slider configurations under standardized in vitro conditions:

- Force loss varied markedly among the tested configurations, with the IO configuration showing the highest resistance to sliding and the BB/TB configurations generally showing lower force loss.
- Configurations with lower force loss generally permitted greater simulated mesial displacement, whereas more constrained configurations showed smaller sagittal movement.
- Translational and rotational side effects differed between configurations, indicating an influence of guide bar geometry and coupling characteristics on the three-dimensional mechanical response.
- The force source (elastic chain vs. closed coil spring) primarily affected force loss, whereas its influence on simulated mesial displacement and most side effects was comparatively limited.

Overall, the findings provide preliminary mechanical reference data demonstrating design-dependent differences in force loss, mesialization, and three-dimensional side effects within the standardized experimental setup.

## Figures and Tables

**Table 1:** A list of commercially available mesial sliders with different configurations from various manufacturers and measured dimensions of the guiding bar.

Abbreviation	Slider	Manufacturer	Geometry and Dimensions (Measured) of the Guiding Bar	Connection Between the Slider and the Molar Band
SO	Slider on Minipin	Ortholize GmbH, Nienhagen, Germany	Rounded Ø 1–1.2 mm	one piece
IO	IZE-Slider on Minipin	Ortholize GmbH, Nienhagen, Germany	round-oval 2.05–2.15 × 1.2 mm	one piece
TB	TADMAN-Beneslider	TADMAN GmbH, Gunningen, Germany	beveled rectangular 1.2 × 0.9 mm	slotted, beveled rectangular (Versalock connector)
BB	BENEfit^®^-Beneslider	PSM Medical GmbH, Gunningen, Germany	Rounded Ø 1 mm	inserted, flat rectangular (mesial tube with hook in conventional band)

**Table 2:** Descriptive statistics (median, interquartile range [IQR]) of the force loss, translational and rotational movements for all slider configurations. Calculated clinical parameters (force loss in %, translational movements in mm, rotational crown movements in °) for all eight slider designs across all ten runs per slider.

Slider	Force Loss (in %)	Translational Movements (in mm)			Rotation of the Crown (in °)		
		Sagittal	Transverse	Vertical	Tipping	Torque	Rotation
BB-C	22.5 [2.0]	−2.93 [0.75]	0.51 [0.12]	−0.30 [0.15]	9.84 [2.09]	2.62 [1.48]	−3.49 [0.31]
BB-S	42.8 [9.2]	−3.55 [0.53]	0.71 [0.30]	0.15 [0.42]	5.67 [2.91]	3.65 [2.34]	−7.71 [0.11]
TB-C	16.6 [2.7]	−2.80 [0.32]	0.29 [0.09]	−0.12 [0.04]	3.59 [3.06]	3.32 [0.59]	−1.23 [0.71]
TB-S	21.1 [2.9]	−3.46 [0.08]	0.49 [0.02]	−0.03 [0.01]	3.67 [0.05]	3.92 [0.26]	−1.28 [0.13]
IO-C	88.1 [2.1]	−0.84 [0.05]	0.05 [0.29]	0.08 [0.06]	1.05 [0.02]	−0.97 [0.15]	−2.44 [0.38]
IO-S	89.8 [0.3]	−0.79 [0.02]	0.00 [0.01]	0.11 [0.01]	1.13 [0.02]	−0.77 [0.06]	−2.44 [0.03]
SO-C	31.9 [6.9]	−1.50 [0.18]	0.19 [0.04]	−0.11 [0.04]	−3.48 [0.09]	1.16 [0.16]	−1.67 [0.14]
SO-S	71.9 [8.2]	−0.99 [0.41]	0.27 [0.17]	−0.09 [0.01]	−4.32 [0.39]	1.12 [0.61]	−3.93 [1.99]

**Table 3:** Pairwise comparisons of the average force loss between all slider configurations, with ten runs per slider. The *p*-values are derived from the Dunn post hoc test following the Kruskal-Wallis test with Benjamini-Hochberg correction. Significance levels: **** = *p* < 0.0001, *** = *p* < 0.001, ** = *p* < 0.01, * = *p* < 0.05, ns = not significant.

Slider	BB-C	BB-S	TB-C	TB-S	IO-C	IO-S	SO-C	SO-S
BB-C	-	ns	ns	ns	***	****	ns	**
BB-S		-	***	*	ns	**	ns	ns
TB-C			-	ns	****	****	**	****
TB-S				-	****	****	ns	***
IO-C					-	ns	*	ns
IO-S						-	***	ns
SO-C							-	ns
SO-S								-

**Table 4:** Pairwise comparisons of the maximal mesialization between all slider configurations, with ten runs per slider. The *p*-values are derived from the Dunn post hoc test following the Kruskal-Wallis test with Benjamini-Hochberg correction. Significance levels: **** = *p* < 0.0001, *** = *p* < 0.001, ** = *p* < 0.01, * = *p* < 0.05, ns = not significant.

Slider	BB-C	BB-S	TB-C	TB-S	IO-C	IO-S	SO-C	SO-S
BB-C	-	ns	ns	ns	**	****	ns	**
BB-S		-	ns	ns	****	****	**	****
TB-C			-	ns	**	***	ns	*
TB-S				-	****	****	**	****
IO-C					-	ns	ns	ns
IO-S						-	*	ns
SO-C							-	ns
SO-S								-

**Table 5:** Pairwise comparisons of the maximum transverse translation between all slider configurations at step 200. The *p*-values are derived from the Dunn post hoc test following the Kruskal-Wallis test with Benjamini-Hochberg correction. Significance levels: **** = *p* < 0.0001, *** = *p* < 0.001, ** = *p* < 0.01, * = *p* < 0.05, ns = not significant.

Slider	BB-C	BB-S	TB-C	TB-S	IO-C	IO-S	SO-C	SO-S
BB-C	-	ns	*	ns	****	****	**	*
BB-S		-	**	ns	****	****	****	***
TB-C			-	ns	*	*	ns	ns
TB-S				-	****	****	**	*
IO-C					-	ns	ns	ns
IO-S						-	ns	*
SO-C							-	ns
SO-S								-

**Table 6:** Pairwise comparisons of the maximum vertical translation between all slider configurations at step 200. The *p*-values are derived from the Dunn post hoc test following the Kruskal-Wallis test with Benjamini-Hochberg correction. Significance levels: **** = *p* < 0.0001, *** = *p* < 0.001, ** = *p* < 0.01, * = *p* < 0.05, ns = not significant.

Slider	BB-C	BB-S	TB-C	TB-S	IO-C	IO-S	SO-C	SO-S
BB-C	-	****	ns	***	****	****	*	*
BB-S		-	**	ns	ns	ns	*	*
TB-C			-	*	***	****	ns	ns
TB-S				-	ns	ns	ns	ns
IO-C					-	ns	**	**
IO-S						-	**	**
SO-C							-	ns
SO-S								-

**Table 7:** Pairwise comparisons of the maximum crown inclination in the mesio-distal direction between all slider configurations at step 200. The *p*-values are derived from the Dunn post hoc test following the Kruskal-Wallis test with Benjamini-Hochberg correction. Significance levels: **** = *p* < 0.0001, *** = *p* < 0.001, ** = *p* < 0.01, * = *p* < 0.05, ns = not significant.

Slider	BB-C	BB-S	TB-C	TB-S	IO-C	IO-S	SO-C	SO-S
BB-C	-	ns	ns	*	****	***	****	****
BB-S		-	ns	ns	**	*	****	****
TB-C			-	ns	*	ns	***	****
TB-S				-	*	ns	***	****
IO-C					-	ns	ns	ns
IO-S						-	ns	*
SO-C							-	ns
SO-S								-

**Table 8:** Pairwise comparisons of the maximum crown torque in the bucco-palatal direction between all slider configurations at step 200. The *p*-values are derived from the Dunn post hoc test following the Kruskal-Wallis test with Benjamini-Hochberg correction. Significance levels: **** = *p* < 0.0001, *** = *p* < 0.001, ** = *p* < 0.01, * = *p* < 0.05, ns = not significant.

Slider	BB-C	BB-S	TB-C	TB-S	IO-C	IO-S	SO-C	SO-S
BB-C	-	ns	ns	ns	****	**	ns	ns
BB-S		-	ns	ns	****	****	**	**
TB-C			-	ns	****	***	*	*
TB-S				-	****	****	***	***
IO-C					-	ns	*	*
IO-S						-	ns	ns
SO-C							-	ns
SO-S								-

**Table 9:** Pairwise comparisons of the maximum mesio-distal crown rotation between all slider configurations at step 200. The *p*-values are derived from the Dunn post hoc test following the Kruskal-Wallis test with Benjamini-Hochberg correction. Significance levels: **** = *p* < 0.0001, *** = *p* < 0.001, ** = *p* < 0.01, * = *p* < 0.05, ns = not significant.

Slider	BB-C	BB-S	TB-C	TB-S	IO-C	IO-S	SO-C	SO-S
BB-C	-	ns	****	***	ns	ns	**	ns
BB-S		-	****	****	**	**	****	*
TB-C			-	ns	**	**	ns	***
TB-S				-	*	*	ns	**
IO-C					-	ns	ns	ns
IO-S						-	ns	ns
SO-C							-	*
SO-S								-

## Data Availability

The datasets generated and/or analyzed during the current study are available from the corresponding author on reasonable request.

## Abbreviations

The following abbreviations are used in this manuscript:

TAD	Temporary anchorage devices
OMSS	Orthodontic Measurement and Simulation System
BB	BENEfit^®^-Beneslider
TB	TADMAN-Slider
IO	IZE-Slider on Minipin
SO	Slider on Minipin
-S	Pull Spring
-C	Elastic Chain
